# Is it sufficient to evaluate bone marrow involvement in newly diagnosed lymphomas using ^18^F-FDG PET/CT and/or routine iliac crest biopsy? A new approach of PET/CT-guided targeted bone marrow biopsy

**DOI:** 10.1186/s12885-018-5104-0

**Published:** 2018-11-29

**Authors:** Bing Hao, Long Zhao, Na-na Luo, Dan Ruan, Yi-zhen Pang, Wei Guo, Hao Fu, Xiu-yu Guo, Zuo-ming Luo, Jing Wu, Hao-jun Chen, Hua Wu, Long Sun

**Affiliations:** Department of Nuclear Medicine & Minnan PET Center, Xiamen Cancer Hospital, The First Affiliated Hospital of Xiamen University, 55 Zhenhai Rd, Xiamen, 361003 Fujian China

**Keywords:** Lymphomas, Bone marrow involvement, ^18^F-FDG PET/CT, Bone marrow biopsy

## Abstract

**Background:**

To investigate whether PET/CT-guided bone marrow biopsy adds complementary information for evaluation of bone marrow involvement (BMI) in newly diagnosed lymphomas.

**Methods:**

Patients with newly diagnosed lymphomas that received both ^18^F-FDG PET/CT and bone marrow biopsy (BMB) were included in this retrospective study. PET/CT classification of bone lesions was classified as isolated, multifocal (2 lesions or more), diffuse (homogeneous uptake of the entire axial skeleton), or negative. BMBs included PET/CT-guided targeted BMB and/or the routine unilateral iliac crest biopsy. Of 34 patients with focal lesions on PET/CT scan, 30 received both PET/CT-guided targeted BMB and iliac crest biopsy, and 4 patients received targeted biopsy without iliac crest biopsy. The final diagnosis of BMI depends on BMB results.

**Results:**

A total of 299 patients with lymphomas were included. PET/CT classification of bone lesions was isolated (16/5.4%), multifocal (67/22.4%), diffuse (52/17.4%), and negative (164/54.8%). If only positive iliac crest biopsy was considered as the reference standard, the sensitivity of ^18^F-FDG PET/CT for identifying focal and diffuse BMI was 48 and 56%, respectively, and the respective specificities were 70 and 83%. Three of 30 patients (10.0%) with focal lesions on PET/CT were confirmed to be false-positive by targeted BMB, and 25 of 30 patients (83.3%) with focal lesions on PET/CT were confirmed as false-negative by iliac crest biopsy.

**Conclusion:**

It is insufficient to evaluate BMI in newly diagnosed lymphomas using both ^18^F-FDG PET/CT and routine iliac crest biopsy. ^18^F-FDG PET/CT imaging should be performed before BMB. In focal bone lesions, PET/CT-guided targeted BMB may complement the results of possible false-positive PET/CT and false-negative iliac crest biopsy findings. However, in diffuse and negative lesions, iliac crest biopsy cannot be safely omitted.

## Background

Lymphomas are malignant neoplasms characterized by the proliferation of cells native to the lymphoid tissue. In 2015, an estimated 88,200 people will be diagnosed with lymphomas, and there will be approximately 52,100 deaths due to the disease in China [1]. Lymphoma subtype and stage affect therapeutic decisions and prognosis. Thus, accurate assessment of lymphoma patients at initial presentation is necessary for good outcomes.

Bone marrow (BM) is an important extent site, which occurs in 5–15% of Hodgkin lymphomas (HLs), and 20–40% of non-Hodgkin lymphomas (NHLs), depending on the histological subtype [2, 3].The evaluation of BM status is of critical importance in newly diagnosed lymphomas, as it is stage, diagnosis, and if prolonged chemotherapy or radiotherapy may be required.

According to the National Comprehensive Cancer Network (NCCN) guidelines for HL and NHL, routine iliac crest biopsy is essential in all lymphomas where treatment is considered, and is the gold standard for bone marrow staging. Unfortunately, results are subject to sampling error, especially focal bone lesions, which may lead to false-negative iliac crest biopsy results. Previous studies have demonstrate bilateral core biopsies can improves the diagnostic yield, ranging between 10 and 60% [4–6], however, an additional biopsy means more risk of complications such as bleeding, pain, and anxiety [7, 8].

There is debate about whether ^18^F-FDG PET/CT scanning can replace routine iliac crest biopsy [9–13]. The Lugano Classification [14] has noted that ^18^F-FDG PET/CT is highly sensitive for BMI which may complement the results of routine iliac crest biopsy, and that is has potential for accurate evaluation of BMI in HL and diffuse large B-cell lymphoma (DLBCL). El-Galaly et al. [15] concluded that routine iliac crest biopsy is not required for evaluation of HL if ^18^F-FDG PET/CT is performed. However, the reference standard according to iliac crest biopsy for true BMI has been questioned [16], since other diseases such as benign conditions or other malignancies can also generate false-positive PET/CT findings [17, 18]. Lim et al. [19] reported that iliac crest biopsy is only needed for DLBCL if the PET/CT finding is negative. The use of PET/CT for evaluation of other subtypes is also controversial. Nevertheless, false-positive PET/CT and false-negative routine iliac crest biopsy results will alter the bone staging of lymphomas. Thus, positive PET/CT findings require histological confirmation, with targeted biopsy preferred to other methods [20, 21].

In our previous study [22], PET/CT-guided targeted BMB was shown to be a safe and effective technique for the evaluation of advanced lung cancer with metastatic bone tumors. However, there are no studies examining the utility of PET/CT-guided targeted BMB, rather than iliac crest biopsy, for bone staging in newly diagnosed lymphomas. The aim of this study was to investigate whether PET/CT-guided targeted BMB adds complementary information for the evaluation of BMI in newly diagnosed lymphomas.

## Methods

### Patients

We retrospectively reviewed the records of patients with a diagnosis of lymphoma treated at out center from January 2012 to January 2018. Patients were identified through the electronic medical records and pathological reporting systems. Patients were included in this study if ^18^F-FDG PET/CT-guided targeted BMB or iliac crest biopsy were performed as part of the initial clinical lymphoma staging, and no other malignancy was present.

Patients did not receive chemotherapy prior to ^18^F-FDG PET/CT scanning, and PET/CT scans were performed within 7 days before or after BMB. Patients were staged according to the Ann Arbor classification. BMI was confirmed based on histological evaluation of biopsy results.

### PET/CT technique and imaging

Patients were asked to fast for 4–6 h before ^18^F-FDG PET/CT scanning. Blood glucose levels were confirmed to be within a maximum value of 120 mg/dL. Intravenous injection of the ^18^F-FDG tracer was administered at a dosage of 0.1–0.2 mCi/kg. Thereafter, the patient rested for 20 min and was encouraged to drink water. A low-dose CT scan from the head to the proximal femur was performed first, with parameters of 110 kV, 110 mA, a tube rotation time of 0.5 s, and 3.3-mm section thickness, which was matched to the PET section thickness. The second scan was immediately followed by a PET scan of the identical transverse field of view. Emission time was acquired for 3 min per table position.

Data acquisition by an integrated PET/CT system (Discovery STE; GE Medical Systems, Milwaukee, WI, USA) was performed within 60 min of tracer injection. PET/CT image data sets were reconstructed iteratively by applying the CT data for attenuation correction, and co-registered images were displayed on an ADW4.3 or ADW4.6 workstation.

PET/CT images were carefully reviewed by 2 senior nuclear medicine physicians. Most indolent lymphomas demonstrate an increased ^18^F-FDG uptake, but the degree of ^18^F-FDG uptake is lower than aggressive lymphomas. If used the positive finding as the uptake over than liver, it may underestimate BMI of indolent lymphomas. ^18^F-FDG uptake of some indolent lymphomas actually was lower that of liver, especially in diffuse BMI.

Positive PET/CT findings were based on the visual assessment of ^18^F-FDG uptake in the involved sites relative to that of the mediastinum. PET/CT-assessed bone lesions were characterized as isolated, multifocal, diffuse, and negative. PET/CT-guided targeted BMB was performed to determinate the diagnosis and bone staging of lymphomas.

### BMB

Two methods of BMB were performed: 1) routine unilateral iliac crest biopsy and 2) PET/CT-guided targeted bone marrow biopsy.

Unilateral iliac crest BMB was routinely in newly diagnosed lymphoma patients performed before any treatment was administered. One sample was obtained for each patient, and lengths of the samples were 1.0 cm.

PET/CT-guided targeted BMB was performed by 1 board certified nuclear medicine physician using the same PET/CT scanner on a separate day to reduce the radiation dose to the operator.^18^F-FDG PET/CT fused images were used to determine the appropriate puncture site with high FDG uptake, and the biopsy needle was introduced stepwise under fused PET/CT image and CT guidance. An 11G (BMN-B, SA Medical & Plastic Instruments Co., Ltd., Shanghai, China) biopsy needle was used. One or two samples were obtained for each patient, and the lengths of samples all were 1.5 or 2.2 cm.

All patients were kept for observation for at least 2 h after the biopsy procedure. The BMB specimens were analyzed by morphological and immunohistochemical studies. The pathological results of all BMBs were validated by review of the individual pathology reports.

### Reference standard

Three reference standards for true BMI were used: 1) the iliac crest biopsy; 2) both focal skeletal PET/CT lesions and positive iliac crest biopsy; 3) PET/CT-guided targeted BMB.

### Statistical analysis and ethics

Median and range were reported for continuous variables, and counts and percentages for categorical variables. Clopper-Pearson exact confidence limits were calculated for sensitivity, specificity, positive predictive value (PPV), negative predictive value (NPV), and accuracy. Differences between PET/CT-guided targeted BMB and routine BMB were analyzed using Fisher exact test. All statistical analysis was performed using SPSS, version 23, and MedCalc, version 12.2.1 software. The *P* value for statistical significance was set to *P* < .05. This retrospective evaluation of collected data was approved by the ethics committee of our institution. All patients gave their written informed consent prior to any procedures performed.

## Results

### Inclusion criteria

A total of 317 patients with lymphomas were identified and screened for eligibility. Twelve patients did not have an iliac crest biopsy in the staging workup, and 6 patients had another coexisting malignancy, and were thus excluded. Thus, 299 patients met the inclusion criteria and were included in the analysis. Of 299 patients identified, 295 patients received an iliac crest biopsy, and 4 patients had only a targeted biopsy. Of the 295 patients received an iliac crest biopsy, 30 patients with focal lesions received both a targeted biopsy and the iliac crest biopsy. Most of the suspicious lymphoma patients will be recommended for PET/CT examination to further diagnosis and differential diagnosis. It is difficult to get definite lymphomas subtypes diagnosis before PET/CT examination in daily clinical practice.

**Fig. 1:**
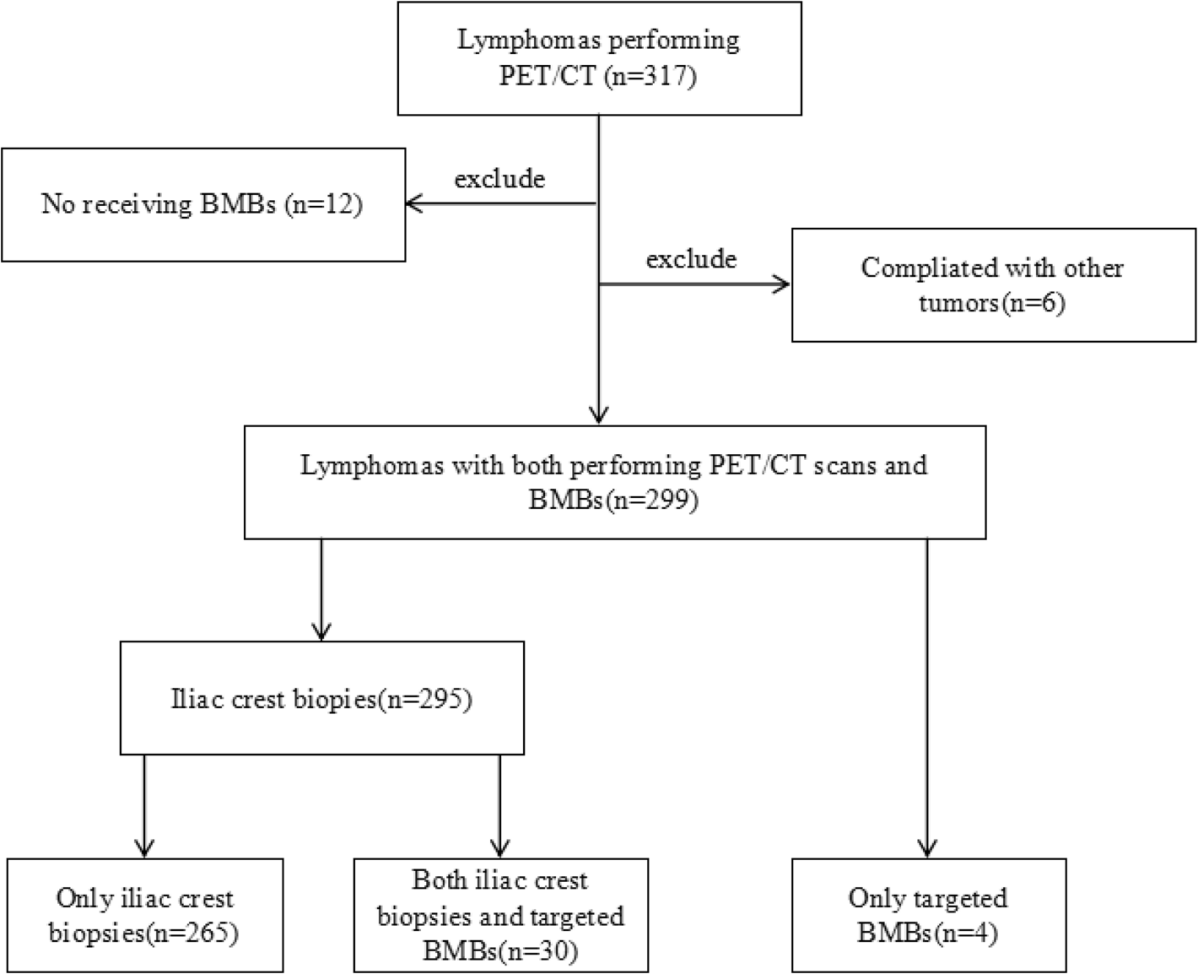
Study flow diagram

Study flow diagram is shown in Fig. 1.

### Clinicopathological characteristics

The median age of patients in the study was 54.8 years (range, 4–91 years), and the male: female ratio was 1:5. The lymphoma subtypes were classified as HL, low-grade, high-grade, NHL, unclassification, Richter transformation and others. Patient characteristic are summarized in the Table 1.

**Table 1:** Clinicopathologic Characteristics of the Study Cohort (*n* = 299)

Characteristic	No. of patients	%
Study subject	299	
Male: Female ratio	1.5	
Age, years		
median	54.8	
range	4–91	
PET-assessed skeletal pattern		
Focal, isolated	16	5.4
Focal, multiple	67	22.4
Diffuse	52	17.4
Negative	164	54.8
Subtypes of lymphoma		
Hodgkin lymphoma^a^	25	8.4
Low-grade Non-Hodgkin lymphoma^b^	68	22.7
High-grade Non-Hodgkin lymphoma^c^	170	56.9
Unclassification^d^	17	5.7
Richter transformation	5	1.7
Others	14	4.6

Abbreviations: *NLP* Nodular lymphocyte predominant, *NS* Nodular sclerosis lymphomas, *MC* Mixed cellularity, *LR* Lymphocyte-rich, *DLBC* Diffuse large B cell, *PTC* Peripheral T cell, *BL* Burkitt lymphoma, *ACL* Anaplastic large cell, *MZ* Marginal zone, *MC* Mantle cell, *MALT* Mucosa-associated lymphoid tissue, *SLL/CLL* Chronic lymphocytic leukemia/small lymphocytic lymphoma

^a^Hodgkin lymphoma included 3 NLP, 10 NS, 8 MC, 4 LR

^b^Low-grade Non-Hodgkin lymphoma included 24 Follicular,10 MZ, 13 MC, 9 MALT, 12 SLL/CLL

^c^High-grade Non-Hodgkin lymphoma included 129 DLBC, 8 PTC, 2 BL, 5 ACL, 2 Lymphoblastic, 25 NK-T

^d^Unclassification included 8 B-cell lymphomas, 8 T-cell lymphomas

### PET/CT classification of bone lesions in newly diagnosed lymphomas

Abnormal skeletal FDG uptake was present in 135 patients (45.2%). Eighty-three of 135 had focal lesions (isolated, 16; multifocal, 67). Of the 83 patients, 16 had positive iliac crest biopsies; 52 of 135 had diffusely homogeneous FDG uptake. Of the 52 patients, 22 had positive iliac crest biopsies involving most of the axial skeleton. Negative skeletal FDG uptake was present in 164 patients (54.8%), of whom 17 had positive iliac crest biopsies. PET/CT data are summarized in Table 2.

**Table 2:** Concordance Between Bone Marrow Biopsy and PET/CT Findings for Evaluation of Bone Marrow Disease (*n* = 295)

	Bone marrow involvement		Total
	+	–	
PET focal bone lesions	16	63	79
PET diffuse bone lesions	22	30	52
PET negative	17	147	164
Total	55	240	295

### Diagnostic performance of ^18^F-FDG PET/CT and iliac crest biopsy for determination of BMI

Results of the diagnostic performance of ^18^F-FDG PET/CT and iliac crest biopsy for evaluating BMI are shown in Table 3. If only positive iliac crest biopsy was considered the reference standard for true BMI, the sensitivity and specificity of ^18^F-FDG PET/CT for detection of focal bone lesions were 48% (95% confidence interval [CI]: 31 to 66%) and 70% (95% CI: 63 to 76%), respectively. The PPV and NPV were 20% (95% CI: 12 to 31%) and 90% (95% CI: 84 to 94%), respectively. The overall diagnostic accuracy (the proportion of true-positive and true-negative results over the total number of tests performed) was 67% (95% CI: 61 to 73%). For diffuse lesions, the sensitivity and specificity were 56% (95% CI: 40 to 72%) and 83% (95% CI: 77 to 88%), respectively. The PPV and NPV were 42% (95% CI: 30 to 57%) and 90% (95% CI: 84 to 90%), respectively. The overall diagnostic accuracy was 78% (95% CI: 72 to 83%).

**Table 3:** Sensitivity, Specificity, PPV, NPV, and Accuracy of the Iliac Crest Biopsy and PET/CT for Detection of Bone Marrow Disease in the 295 patients

Diagnostic Modality	%	95%CI		%	95%CI
	Bone Disease Defined Only by Positive Iliac crest biopsy (*n* = 55)				
Iliac crest biopsy					
Focal			Diffuse		
Sensitivity	N/A^a^		Sensitivity	N/A^a^	
Specificity	N/A^a^		Specificity	N/A^a^	
PPV	N/A^a^		PPV	N/A^a^	
NPV	N/A^a^		NPV	N/A^a^	
Accuracy	N/A^a^		Accuracy	N/A^a^	
PET/CT					
Focal			Diffuse		
Sensitivity	48	31 to 66	Sensitivity	56	40 to 72
Specificity	70	63 to 76	Specificity	83	77 to 88
PPV	20	12 to 31	PPV	42	30 to 57
NPV	90	84 to 94	NPV	90	84 to 90
Accuracy	67	61 to 73	Accuracy	78	72 to 83
	Bone Disease Defined Only by Positive Iliac crest biopsy and/or FDG-avid bone lesion (*n* = 148)				
Iliac crest biopsy					
Focal			Diffuse		
Sensitivity	34	25 to 45	Sensitivity	57	44 to 68
Specificity	N/A^b^		Specificity	N/A^b^	
PPV	N/A^b^		PPV	N/A^b^	
NPV	70	63 to 76	NPV	83	77 to 88
Accuracy	74	68 to 79	Accuracy	86	81 to 90
PET/CT					
Focal			Diffuse		
Sensitivity	82^‡^	71 to 88	Sensitivity	75^‡^	63 to 85
Specificity	N/A^b^		Specificity	N/A^b^	
PPV	N/A^b^		PPV	N/A^b^	
NPV	90^‡^	84 to 94	NPV	90^‡^	84 to 94
Accuracy	93^‡^	89 to 96	Accuracy	92^‡^	88 to 95

Abbreviations: *PET* Positron emission tomography, *CT* Computed tomography, *N/A* Not applicable, *NPV* Negative predictive value, *PPV* Positive predictive value

^‡^*P* < 0.05 for difference between the routine iliac crest biopsy and PET/CT for detection of bone marrow disease

^a^N/A because iliac crest biopsy is considered the gold standard in this analysis

^b^N/A because of missing reference for true positive

If focal skeletal PET/CT lesions and positive iliac crest biopsy were both considered as the reference standard for true BMI, the sensitivity of focal bone lesions was 82% (95% CI: 71 to 88%) for PET/CT versus 34% (95% CI: 25 to 45%) for iliac crest biopsy. The NPVs were 90% (95% CI: 84 to 94%) for PET/CT versus 70% (95% CI: 63 to 76%) for iliac crest biopsy. The overall diagnostic accuracy was 93% (95% CI: 89 to 96%) for PET/CT versus 74% (95% CI: 68 to 79%) for iliac crest biopsy. As for diffuse lesions, the sensitivity was 75% (95% CI: 63 to 85%) for PET/CT versus 57% (95% CI: 44 to 68%) for iliac crest biopsy. The NPVs were 90% (95% CI: 84 to 94%) for PET/CT versus 83% (95% CI: 77 to 88%) for iliac crest biopsy. The overall diagnostic accuracy was 92% (95% CI: 89 to 95%) for PET/CT versus 86% (95% CI: 81 to 90%) for iliac crest biopsy.

### PET/CT findings of indolent lymphomas

The main subtypes of indolent lymphomas were follicular, marginal zone, mucosa-associated lymphoid tissue (MALT), small lymphocytic lymphoma/chronic lymphocytic leukemia (SLL/CLL) and mantle cell lymphomas. The ^18^F-FDG avidity of this group of lymphomas was demonstrated in Table 4.

**Table 4:** ^18^F-FDG avidity of Indolent lymphomas

Indolent lymphomas subtype	N	^18^F-FDG-avid	Negative	%^18^F-FDG-avid	Focal/Diffuse/Negative bone lesions
Follicular	24	20	4	83.3	2/4/18
Marginal zone	10	9	1	90	1/6/3
MALT	9	6	3	66.7	0/0/9
SLL/CLL	12	9	3	75	2/4/6
Mantle cell	13	13	0	100	0/3/10

Abbreviations: *MALT* Mucosa-associated lymphoid tissue, *SLL/CLL* Small lymphocytic lymphoma/chronic lymphocytic leukemia

### Differences of bone staging between PET/CT-guided targeted BMB and iliac crest biopsy for focal bone lesions

A comparison of the diagnostic performance between PET/CT-guided targeted BMB and iliac crest biopsy for the assessment of focal BM disease in the 30 patients is shown in Table 5. The diagnostic ability of targeted BMB was superior to that of iliac crest biopsy (sensitivity of 90.0% versus 16.7%, respectively, *P* < 0.05). Three of 30 patients with a negative targeted biopsy were confirmed to have false-positive PET/CT findings, and the pathological results included 1 eosinophilic granuloma with fibrosis, 1 myelodysplastic syndrome (MDS), and 1 active bone marrow hyperplasia. Twenty-five of 30 patients with focal lesions on PET/CT scan were confirmed to have false-negative results of iliac crest biopsies. In addition, in 4 patients who had a targeted BMB without an iliac crest biopsy, the diagnosis and bone staging was established in a single procedure.

**Table 5:** A Comparison of the Diagnostic Performance Between PET/CT-guided Targeted BMB and Iliac Crest Biopsy for the Assessment of Focal Bone marrow disease on PET/CT scan in the 30 patients

	Pathological results		Total	*P*
	+	–		
Target bone biopsy(*n* = 30)	27 (90.0%)	3^a^ (10.0%)	30	< 0.05^‡^
Routine iliac crest biopsy(*n* = 30)	5 (16.7%)	25 (83.3%)	30	

^a^Negative results include one Eosinophilic granuloma with fibrosis, one myelodysplastic syndrome (MDS) and one active bone marrow hyperplasia

^‡^ Fisher exact test

### BMB related inflammatory response and complications

Forty-eight patients with focal lesions received PET/CT scan after BMB. In those patients, only 4(8.3%) patients had slightly ^18^F-FDG uptake in the puncture site, the degree of ^18^F-FDG uptake of puncture site is obviously lower than that of lesions of BMI. The average time to perform a biopsy was 30 min. Slight bleeding and pain occurred in almost all patients who underwent a biopsy, but no serious complications were noted (Table 6).

**Table 6:** Analysis of the 34 PET/CT-guided targeted BMBs for PET-assessed staging IV patients

Biopsy site	No. of biopsies	Biopsy procedure			Diagnosis		Bone morpholovic abnormalities on CT findings	
		Success	Complications		Subtype^a^	Other^b^	No. of Yes	No. of No
			Slight pain	Slight bleeding				
Ilium	22	22	15	15	19	3	15	8
Sacrum	6	6	5	4	6	0	1	5
Ischium	3	3	3	3	3	0	1	1
Rib	1	1	0	0	1	0	1	0
Femur	2	2	2	2	2	0	1	1
Total	34	34	25	24	31	3	19	15

^a^Subtype means definite subtype diagnosis, such as diffuse large B cell lymphoma

^b^Other include one Eosinophilic granuloma with fibrosis, one myelodysplastic syndrome (MDS) and one active bone marrow hyperplasia

**Fig. 2:**
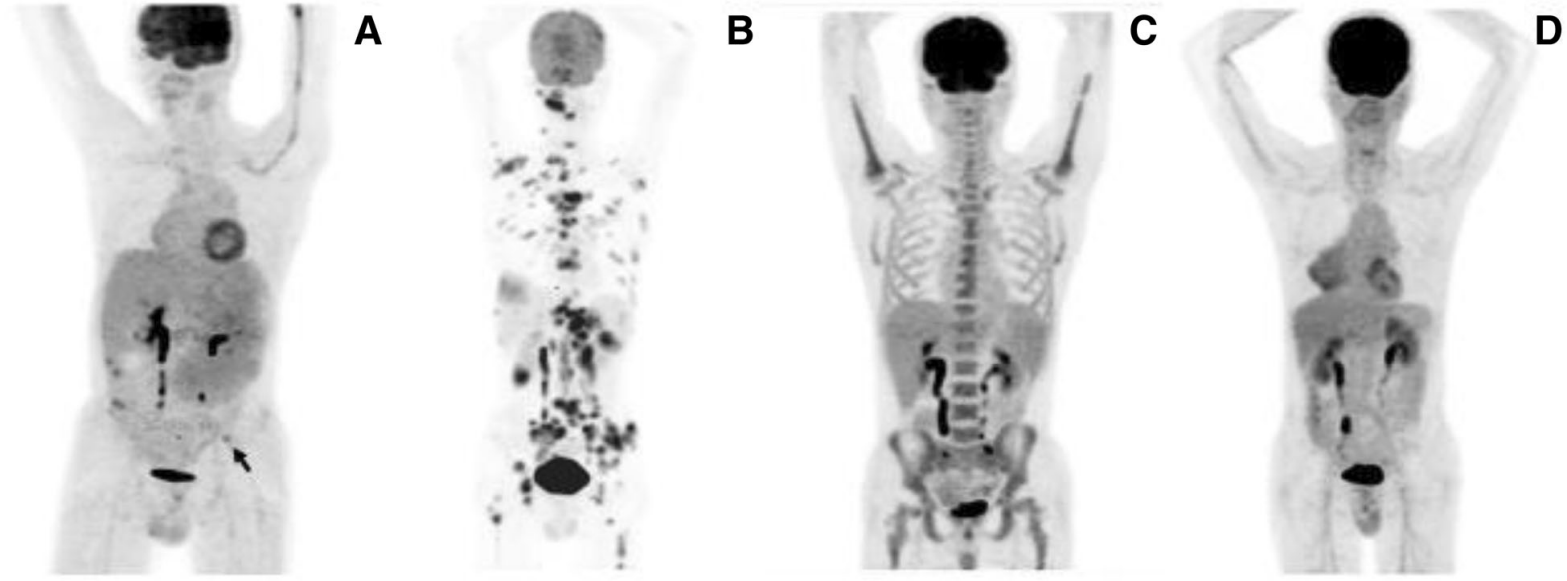
PET/CT classification of bone marrow involvement (maximum intensity projection images, MIP). **a** Isolated lesion in the left ilium (black arrow). **b** Multifocal lesions in bone marrow. **c** Diffuse lesions in axial skeleton. **d** Negative PET findings in bone marrow

**Fig. 3:**
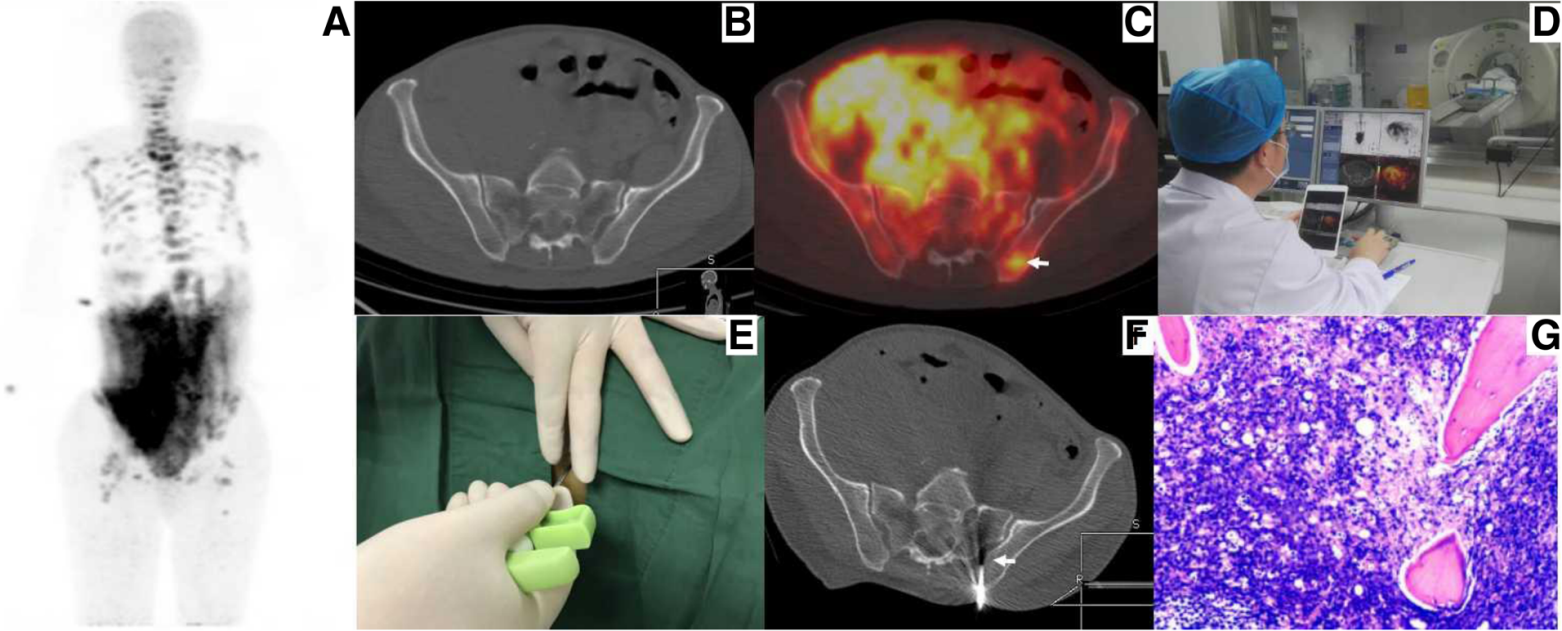
PET/CT-guided targeted bone marrow biopsy procedure. **a-c** ^18^F-FDG PET/CT findings. **a** The MIP image showed multifocal lesions in the region of bone and abdomen. **b** and **c** Axial CT image, axial PET/CT fusion image, showed high ^18^F-FDG uptake within the left ilium (white arrow), while no overt bony change was observed within the corresponding hypermetabolic region. **d** Procedure of biopsy target selection. **e** Puncture process using a 16G bone biopsy needle. **f** CT image showed the biopsy needle positioned within the left ilium lesion (white arrow). **g** Histological examination combined with immunohistochemical result confirmed a diagnosis of NHL (high-grade B cell lymphoma), suggesting further examination with C-MYC, Bcl-2, and Bcl-6 genetic testing

**Fig. 4:**
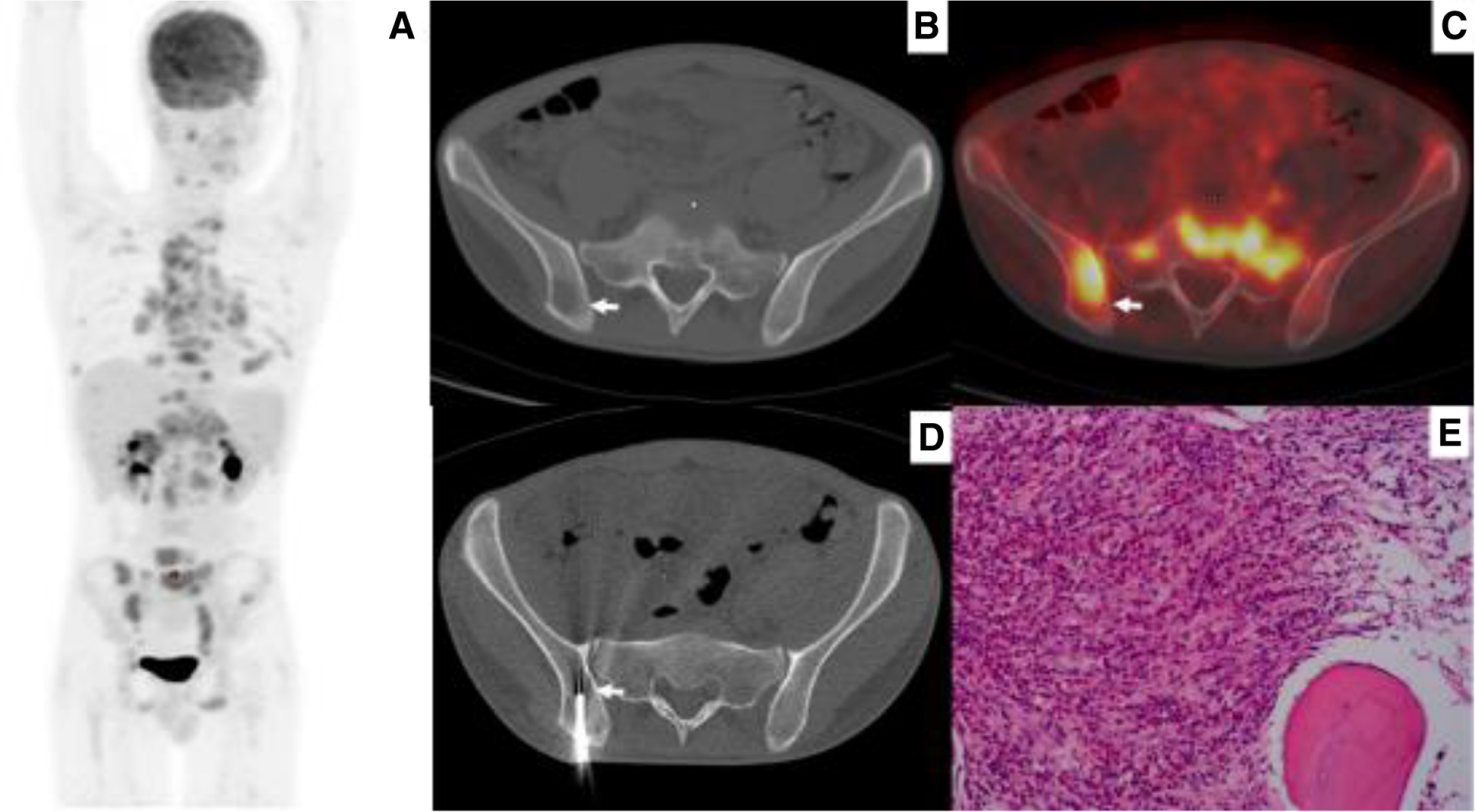
A patient with mixed cell Hodgkin lymphoma, who was suspected of having multifocal bone lesions on the PET/CT image. Targeted biopsy confirmed false-positive PET findings. **a** MIP image showed multifocal bone lesions. **b** and **c** Axial CT image, axial PET/CT fusion image, showed high ^18^F-FDG uptake within the right ilium (white arrow) and osteolytic destruction was observed within the corresponding hypermetabolic region (white arrow). **d** CT image showed the biopsy needle positioned within the right ilium lesion (arrow). **e** Histological examination combined with immunohistochemical result confirmed the diagnosis of eosinophilic granuloma with fibrosis

**Fig. 5:**
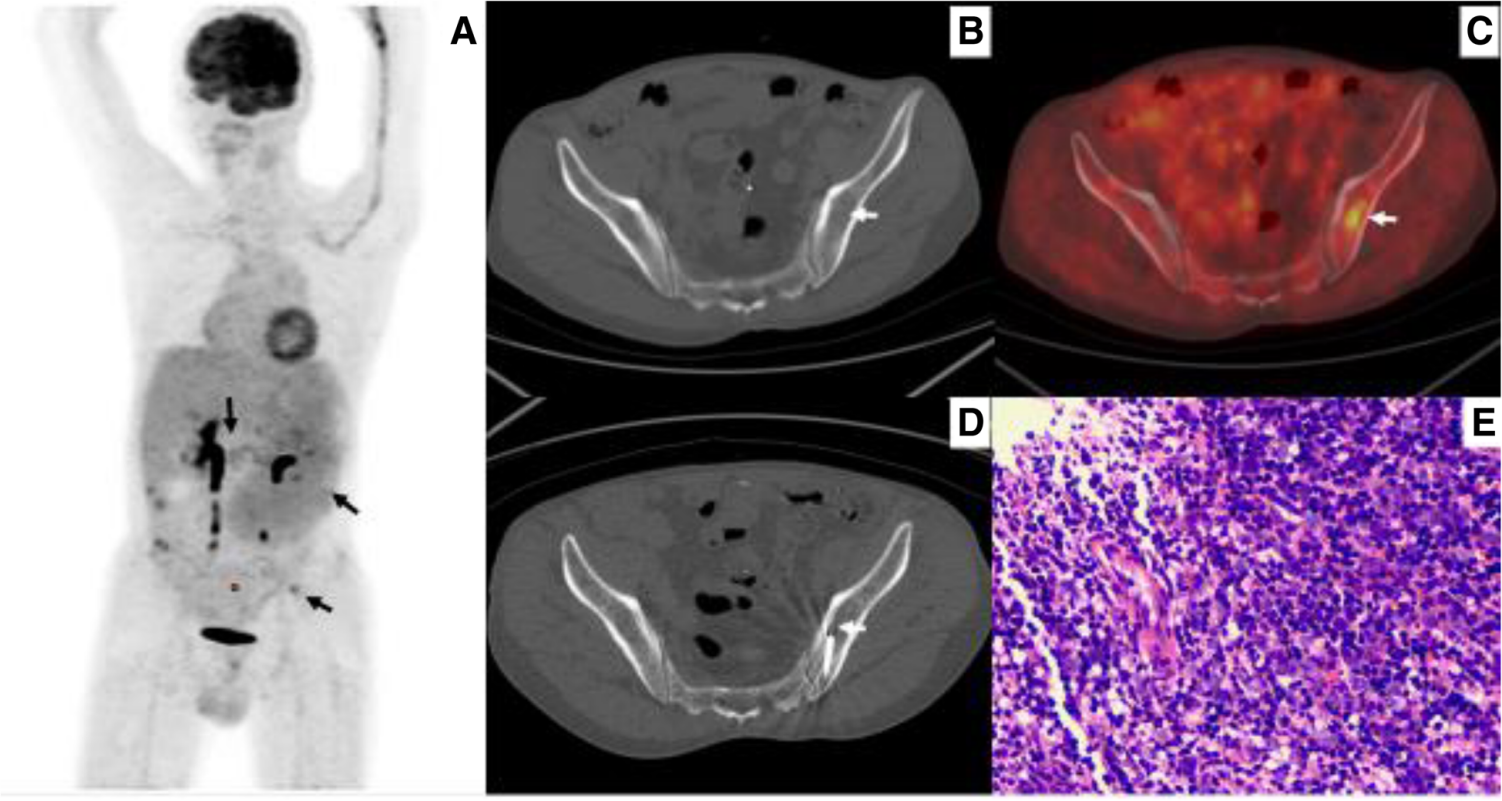
A patient who was suspected of having a lymphoma based on ^18^F-FDGPET/CT findings. Both diagnosis and staging were completed through PET/CT-guided targeted bone marrow biopsy in one procedure. The iliac crest biopsy was negative. **a** MIP image showed high ^18^F-FDG uptake within lesions in multiple retroperitoneal lymph nodes, spleen, and isolated left ilium. **b** and **c** PET/CT image showed high ^18^F-FDG uptake (SUVmax 3.38) within the left ilium, while no abnormality was observed on corresponding CT image (arrow). **d** CT image showed the biopsy needle positioned within the left ilium lesion (arrow). **e** Histological examination combined with immunohistochemical result confirmed a diagnosis of DLBCL

**Fig. 6:**
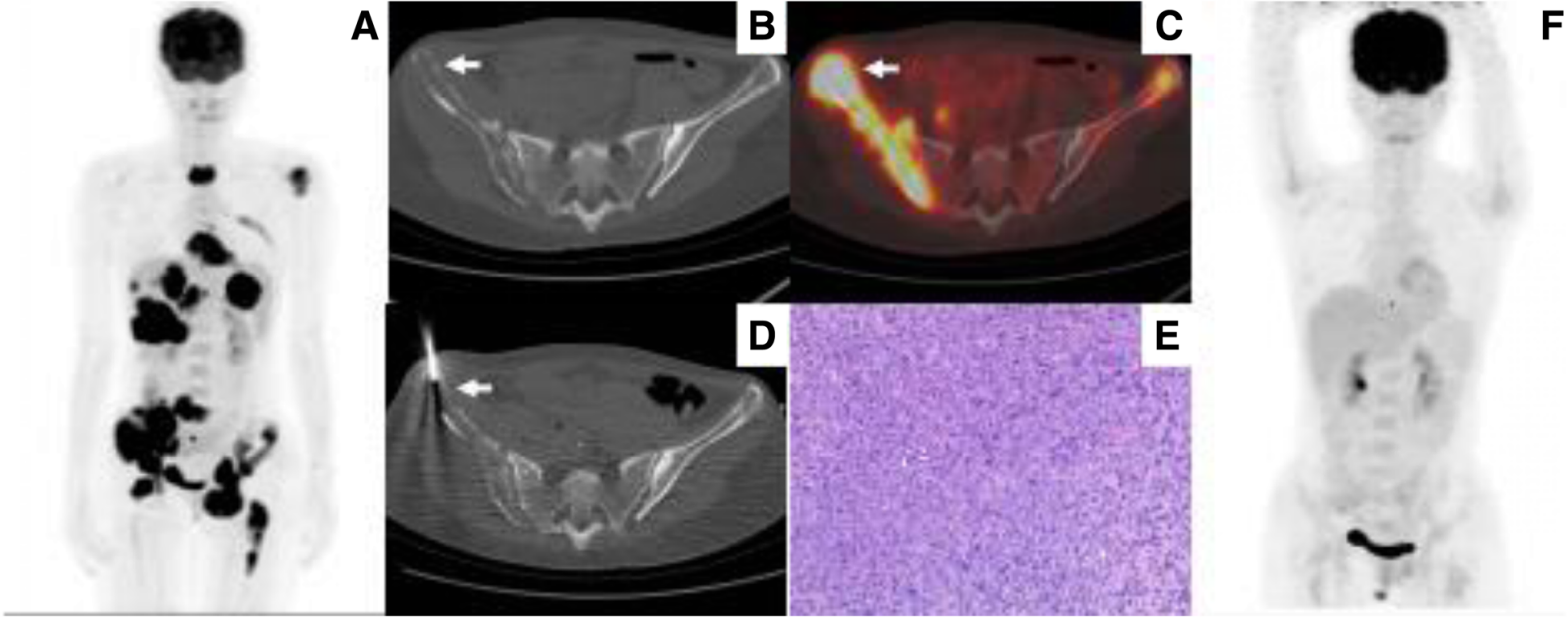
A patient with suspected lymphoma involvement in multiple areas of bone marrow, liver, spleen, and lymph nodes. She received targeted biopsy for diagnosis and staging. **a** MIP image showed high ^18^F-FDG uptake within lesions in the bone marrow, liver, spleen, and lymph nodes. **b** and **c** Axial PET/CT image showed high ^18^F-FDG uptake (SUVmax 17.34) within the left ilium, and abnormality on the corresponding CT image (arrow). **d** CT image showed the biopsy needle positioned within the left ilium lesion (arrow). **e** Histological examination combined with immunohistochemical result confirmed a diagnosis of DLBCL. **f** MIP of follow-up ^18^F-FDG PET/CT at the end of chemotherapy showed no hypermetabolic lesions (complete response)

Representative images are shown in Figs. 2, 3, 4, 5 and 6.

## Discussion

To our knowledge, this study is the first to propose the notion that PET/CT-guided targeted BMB can add pathological confirmation of focal BMI in newly diagnosed lymphomas. Based on the data that ^18^F-FDG PET/CT overcomes deficiencies of iliac crest biopsy, targeted BMB has gradually become routine practice at our institution.

^18^F-FDG PET/CT has been widely accepted for the evaluation of focal BMI of HL, without the need for routine iliac crest biopsy [14, 15]. However, its actual diagnostic value is difficult to determine because of scarce target biopsies, and only the iliac crest biopsy as a reference standard for true BMI may erroneously generate false-positive PET/CT and false-negative iliac crest biopsy results. Our results demonstrated that 1 of 2 patients with HL with focal bone lesions on PET/CT scan, together with a negative iliac crest biopsy, confirmed the false-positive PET/CT findings via a targeted biopsy. If all subtypes were included, in the 30 patients in whom a targeted biopsy and an iliac crest biopsy were performed, 3 of 30 patients who had a positive PET/CT findings were shown to have other BM diseases rather than lymphoma BMI by targeted biopsy. Therefore, although ^18^F-FDG PET/CT can easily detect the bone lesions, positive findings also required histological examination, even in cases of HL.

Adequate imaging is a prerequisite for performing a biopsy to establish a diagnosis. Advances in imaging techniques have contributed to the increasing use of image-guided percutaneous biopsy for the diagnosis of primary and secondary bone tumors [23, 24].^18^F-FDG PET/CT is the preferred method for determining the choice of biopsy site, as it is superior to other imaging methods [25]. Compared to a conventional CT-guided biopsy, the dual modality technique provides co-registered information about both the morphology and physiology of the proposed biopsy site, and can offer a target that exhibits increased FDG uptake [26]. In addition, whole body PET/CT allows visualization of the entire marrow and provides better visualization of poorly defined bone abnormalities than other modalities [27, 28]. In our study, 19 of 34 patients had no bone abnormalities on CT and were thus not suitable for CT-guided biopsy, while PET/CT identified an accurate biopsy site. Most importantly, the PET/CT classification of bone lesions of lymphomas is useful for determining the choice of biopsy. The pattern of skeletal FDG uptake was categorized as isolated, multifocal, diffuse, and negative. Our results also demonstrated 5 of 68 patients (7.4%) with focal ^18^F-FDG–avid bone lesions in indolent lymphomas. Individual algorithms for evaluation of BMI should be performed for various bone lesions identified on PET/CT imaging. In general, failure to follow appropriate biopsy procedures may lead to inaccurate bone staging.

Currently, routine iliac crest biopsy is still the standard bone staging method for patients with newly diagnosed lymphomas [29]. However, it is well-known that the procedure is associated with low sensitivity in newly diagnosed lymphomas, especially those with focal BMI. In other words, a negative routine iliac crest biopsy cannot rule out the presence of BMI. Our results showed that PET/CT had a sensitivity of 48% in 79 patients with focal bone lesions. Sampling error, especially outside the iliac crest, may be major factor. Thus, it is preferable to use PET/CT-guided targeted BMB in the case of positive PET/CT findings to easily obtain representative tissue. First, a separate staging procedure adds time, cost, coordination of care, inconvenience, and an additional anesthetic risk [7, 30].In the 34 patients in our study,4 patients were simultaneously confirmed diagnosis and stage of lymphoma only through targeted BMB so that iliac crest biopsy could be avoided. Second, targeted biopsy avoided false-positive PET/CT findings in 3 of 34 patients in our study, which can result in inaccurate staging. Third, targeted biopsy was obviously superior to routine iliac crest biopsy in our study (sensitivity of 90.0% for targeted biopsy vs. 16.7% for iliac crest biopsy), and a false-negative iliac crest biopsy was avoided. In addition, we believe that targeted biopsy is a suitable reference standard to calculate the specificity of bone marrow ^18^F-FDG PET.

PET/CT-guided targeted BMB is a safe and feasible method for bone lesions [27, 31–33]. The preferred choice of puncture site for an individual patient depends on the size and location and local experience and expertise. In our study, the relatively safer puncture site that is suspicious BMI on ^18^F-FDG PET/CT was preferred consideration and no serious complications were noted. Meanwhile, an adequate amount of tissue is required for immunophenotyping and genetic testing to establish a definitive diagnosis of lymphomas [29]. In our study, an adequate amount of tissue was obtained in all 34 patients and definitive subtype diagnosis was determined in 31 of 34 patients. The overall success rate was 91.2%. The remaining two patients were diagnosed with B-cell lymphomas, and didn’t receive immunophenotyping and genetic testing for differential diagnosis due to lack of funds. Thus, our biopsy procedure actually showed a safe, feasible and effective method for diagnosis of lymphomas. When an FDG-avid peripheral lymph node is not present, or in the case of primary bone lymphoma, a targeted BMB is a potential alternative for accurate diagnosis, and complete staging in a single procedure. However, targeted bone marrow biopsy in some localizations may be relatively difficult, such as skull, anterior part of vertebra .etc. The puncture site should be selected in the relatively safe localizations for advoiding serious complication.

Diffuse bone lesions on PET/CT scan are usually defined as homogeneous increased FDG uptake of the entire axial skeleton, and are a well-known phenomenon in patients treated with hematopoietic growth factors [34, 35]. However, the finding is relatively uncommon in newly diagnosed lymphomas [36]. In a study by Admas et al. [37], the incidence of diffuse bone lesions on PET/CT scan was 4.2% (23/542), and the frequency of positive iliac crest biopsies was 55.0% (11/20) in newly diagnosed lymphomas. But our current study reported that the incidences of diffuse skeletal uptake were about 17.4%. The difference in the incidences is due to the different PET positive standard. We considered ^18^F-FDG FDG uptake greater the mediastinum as PET positive standard, while Hugo J.A. Adams refer to ^18^F-FDG FDG uptake greater than liver. Thus, it is explainable why the incidence of diffuse bone marrow FDG uptake in our current study was higher than that of Hugo J.A. Adams^,^s results. This present study showed that ^18^F-FDG PET/CT does not exhibit very good concordance with the results of iliac crest biopsy, and its sensitivity and specificity were only 56 and 83%, respectively. Thus, iliac crest biopsy is more useful for diffuse bone lesions seen on PET/CT scan compared with other modality.

A routine iliac crest biopsy also cannot be safely omitted in the case of negative bone marrow PET/CT findings, except in cases of DLBCL [14, 19].In our study, we did not find positive iliac crest biopsy results if the PET/CT was negative in patients with DLBCL. But in other subtypes of lymphomas, of 164 patients who had negative PET/CT results, 17 had positive iliac crest results, resulting in an overall NPV of 90%. Thus, 10% of the patients would be inaccurate staging if the iliac crest biopsy was not performed.

Some limitations of this study should be acknowledged. First, the sample size of PET/CT-guided targeted BMBs was limited. We tried to retrieve multi-center date to test and verify our results. Second, only ^18^F-FDG PET/CT and iliac crest biopsy were studied, more other modality, such as MRI, laboratory examination, etc. must be considered. Finally, subtypes of lymphomas present differences depending on various PET/CT findings. In regard to these problems, more studies will be followed in the future gradually.

## Conclusion

It is insufficient to evaluate BMI in newly diagnosed lymphomas using both ^18^F-FDG PET/CT and routine iliac crest biopsy. It is recommended that ^18^F-FDG PET/CT imaging be performed before any BMB. In focal lesions identified on ^18^F-FDG PET/CT scan, it is preferable to PET/CT-guided targeted biopsy, because it could not exclude possible false-positive PET/CT findings, even in HL, and can also avoid additional blind biopsies. However, in diffuse and negative lesions identified on ^18^F-FDG PET/CT scan, iliac crest biopsy cannot be safely omitted.

## Abbreviations

95%CI: 95% confidence interval
BM: Bone marrow
BMI: Bone marrow involvement
DLBCL: Diffuse large B-cell lymphoma
HL: Hodgkin lymphoma
MALT: Mucosa-associated lymphoid tissue
MDS: Myelodysplastic syndrome
NCCN: National Comprehensive Cancer Network
NHL: Non-Hodgkin lymphomas
NPV: Negative predictive value
PPV: Positive predictive value
SLL/CLL: Small lymphocytic lymphoma/chronic lymphocytic leukemia
